# Ischemic Stroke Mortality in Type 2 Diabetes in the U.S.: National Trends and Demographic Disparities From 1999 to 2019

**DOI:** 10.1002/edm2.70065

**Published:** 2025-07-13

**Authors:** Muhammad Moiz Nasir, Syed Husain Farhan, Hasan Mushahid, Syeda Ayesha Shah, Muhammad Hamza Shuja, Adam Bilal Khan, Syed Hassaan Ali, Syed Ahmed Farhan, Azeem Hassan, Jawad Ahmed, Mohammad Hamza, Javed Iqbal

**Affiliations:** ^1^ Department of Internal Medicine Dow University of Health Sciences Karachi Pakistan; ^2^ Department of Internal Medicine McLaren Greater Lansing Hospital Michigan Indiana USA; ^3^ National Heart and Lung Institute Imperial College London London UK; ^4^ Department of Internal Medicine Northwest Health—Porter Valparaiso Indiana USA; ^5^ Dow University of Health Sciences Karachi Pakistan; ^6^ Guthrie Medical Group Cortland New York USA; ^7^ Nursing Department Communicable Diseases Centre Hamad Medical Corporation Doha Qatar

**Keywords:** database, diabetes mellitus, observational study, stroke

## Abstract

**Background:**

The pathological changes in the lining of blood vessels associated with diabetes are a well‐established risk factor for stroke, with some studies suggesting a two times increase in risk compared to non‐diabetics.

**Methods:**

Death certificates from the CDC WONDER (Centers for Disease Control and Prevention Wide‐Ranging OnLine Data for Epidemiologic Research) database were examined from 1999 to 2019 for ischemic stroke‐related mortality in patients with type 2 diabetes mellitus (T2DM). Annual percent change (APC) and age‐adjusted mortality rates (AAMRs) per 100,000 persons were calculated and stratified by year, sex, and race/ethnicity.

**Results:**

From 1999 to 2019 there were 18,135 deaths from ischemic stroke in patients with T2DM. The AAMR remained relatively constant from 0.31 in 1999 to 0.32 in 2004, gradually declining to 0.14 in 2014 (APC: −6.74), followed by a rapid increase to 0.44 in 2017 (APC: 53.11). Men showed consistently higher AAMR than women in 1999 (AAMR men: 0.34 vs. women: 0.29) and 2019 (AAMR men: 0.55 vs. women: 0.42). When comparing race, African Americans (AA) presented with a consistently higher AAMR in 1999 (AAMR AA: 0.4 vs. white: 0.29) and in 2019 (AAMR AA: 0.62 vs. white:0.44). Notably, a significant escalation in AAMR occurred from 2014 to 2019, affecting both populations; this trend reached its pinnacle in 2019 (2016 AAMR AA: 0.4 vs. white: 0.26) (2019 AAMR AA: 0.62 vs. white: 0.44).

**Conclusion:**

The findings highlight fluctuating trends in AAMRs with distinct shifts observed after 2014. Noteworthy gender and racial disparities in AAMRs were also evident. The study emphasises the need for ongoing vigilance and focused interventions to address the evolving dynamics of ischaemic stroke‐related mortality in the T2DM population.

## Graphical abstract

A Central illustration showing ischemic stroke mortality trends in T2DM patients (1999–2019), showing a steady decline until 2014, followed by a sharp increase after 2017. Men and African Americans consistently had higher AAMRs.

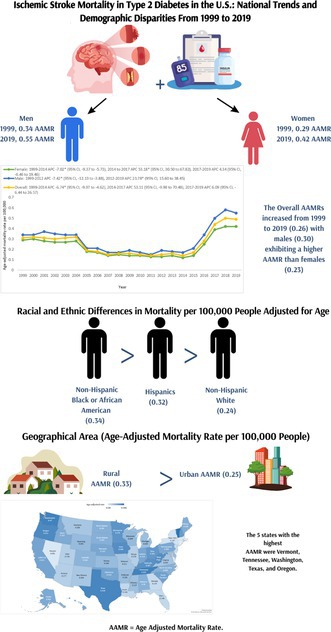

## 1 Introduction

Stroke ranks as the fifth leading cause of death in the United States, accounting for 162,890 fatalities in 2021 [1]. Type 2 diabetes mellitus (T2DM) is well‐established as a significant risk factor for stroke, with studies indicating that individuals with T2DM have nearly a 200% increased likelihood of experiencing a stroke compared to non‐diabetics, even after adjusting for age [2, 3, 4, 5]. In fact, within the diabetic population, 65% of deaths are attributable to cardiovascular diseases (CVD), stroke, or both [3]. Among the two main subtypes of stroke, the prevalence of diabetes is higher in patients with ischemic stroke (33%) than in those with hemorrhagic stroke (26%) [5].

The risk of developing T2DM increases with age across all racial and ethnic groups [6], and this increase is accompanied by a consistent rise in ischemic stroke incidence across all age groups [3, 7]. Studies conducted between 2010 and 2019 have particularly highlighted an elevated stroke risk among African Americans under the age of 55. During this period, African Americans faced a 3–4 times higher risk of death from stroke compared to whites, in both men and women, with the disparity being especially pronounced before the age of 65 [8, 9, 10, 11]. Interestingly, this difference in ischemic stroke risk between African Americans and whites tends to diminish in the elderly population, contrasting with the typically straightforward and linear relationship observed between T2DM and age [3, 7, 11].

To address these age and gender‐specific variations and to develop targeted preventive strategies and management approaches for diverse ethnic populations, we aimed to evaluate demographic and regional differences in ischemic stroke‐related mortality among patients with T2DM in the United States from 1999 to 2019.

## 2 Methods

### 2.1 Study Sample

The CDC WONDER (Centers for Disease Control and Prevention Wide‐Ranging OnLine Data for Epidemiologic Research) database was queried to retrieve death certificate data for ischemic stroke‐related deaths in diabetics from 1999 to 2019. The International Statistical Classification of Diseases and Related Health Problems‐10th Revision (ICD‐10) codes used were as follows: E11 (Non‐insulin‐dependent diabetes mellitus) and I63 (Cerebral infarction). These ICD‐10 codes have been used previously to identify T2DM and ischemic stroke in administrative databases [12, 13]. A review of death certificates from the Multiple Cause‐of‐Death Public Use records was undertaken to identify cases associated with ischemic stroke among individuals with T2DM. Specifically, instances where both ischemic stroke and T2DM were documented on the death certification, either as contributing or underlying causes of death, were singled out for analysis. Our analysis utilized deidentified publicly accessible data available from the CDC WONDER database, obviating the need for ethical committee approval or informed consent.

### 2.2 Data Extraction

Information about population size, year, geographical location of death, demographic details, urban–rural classification, region, and state‐specific data were extracted. People of all ages were included and grouped into 10‐year age brackets. Demographic variables encompassed gender and race/ethnicity. The location of death was categorised into medical facilities (outpatient, emergency room, inpatient, death on arrival, or status unknown), decedent's home, hospice facility, and nursing homes/long‐term care facilities. Race/ethnicity was classified as non‐Hispanic (NH) American Indian or Alaskan Native, NH Asian or Pacific Islander, NH Black or African American, or NH White. The National Center for Health Statistics Urban–Rural Classification Scheme was used to assess the population by urban (large metropolitan area [population ≥ 1 million], medium/small metropolitan area [population 50,000–999,999]) and rural (population < 50,000) counties per the 2013 U.S. census classification [14]. Region was determined based on the U.S. Census Regions, and all four regions were included (Northeast, Midwest, South, and West).

### 2.3 Statistical Analysis

We computed both crude and age‐adjusted mortality rates (AAMRs) per 100,000 individuals from 1999 to 2019. Demographic factors, including year, gender, race/ethnicity, state, and urban–rural status, were considered with 95% confidence intervals (CIs). Crude mortality rates were computed by dividing the total of deaths related to ischemic stroke by the respective U.S. population for each year, while AAMRs were derived by standardising against the population of the U.S. in 2000 [15]. To evaluate annual trends in mortality associated with ischemic stroke, we utilized the Joinpoint Regression Program (version 4.9.0.0, National Cancer Institute) which was used for the determination of the annual percent change (APC) and corresponding 95% CI in the AAMR [16]. APCs were classified as increasing or decreasing depending on whether the mortality change slope significantly differed from zero, as assessed through a two‐tailed t‐test with a significance threshold of *p* < 0.05.

## 3 Results

Between 1999 and 2019, there were 18,135 deaths related to ischemic stroke in individuals with T2DM. Details about the location of death were available for 17,499 of these cases. Among these, 45% occurred in medical facilities, 30.5% in nursing homes or long‐term care facilities, 4.9% in hospices, and 19.7% at home (Table S1).

**FIGURE 1:**
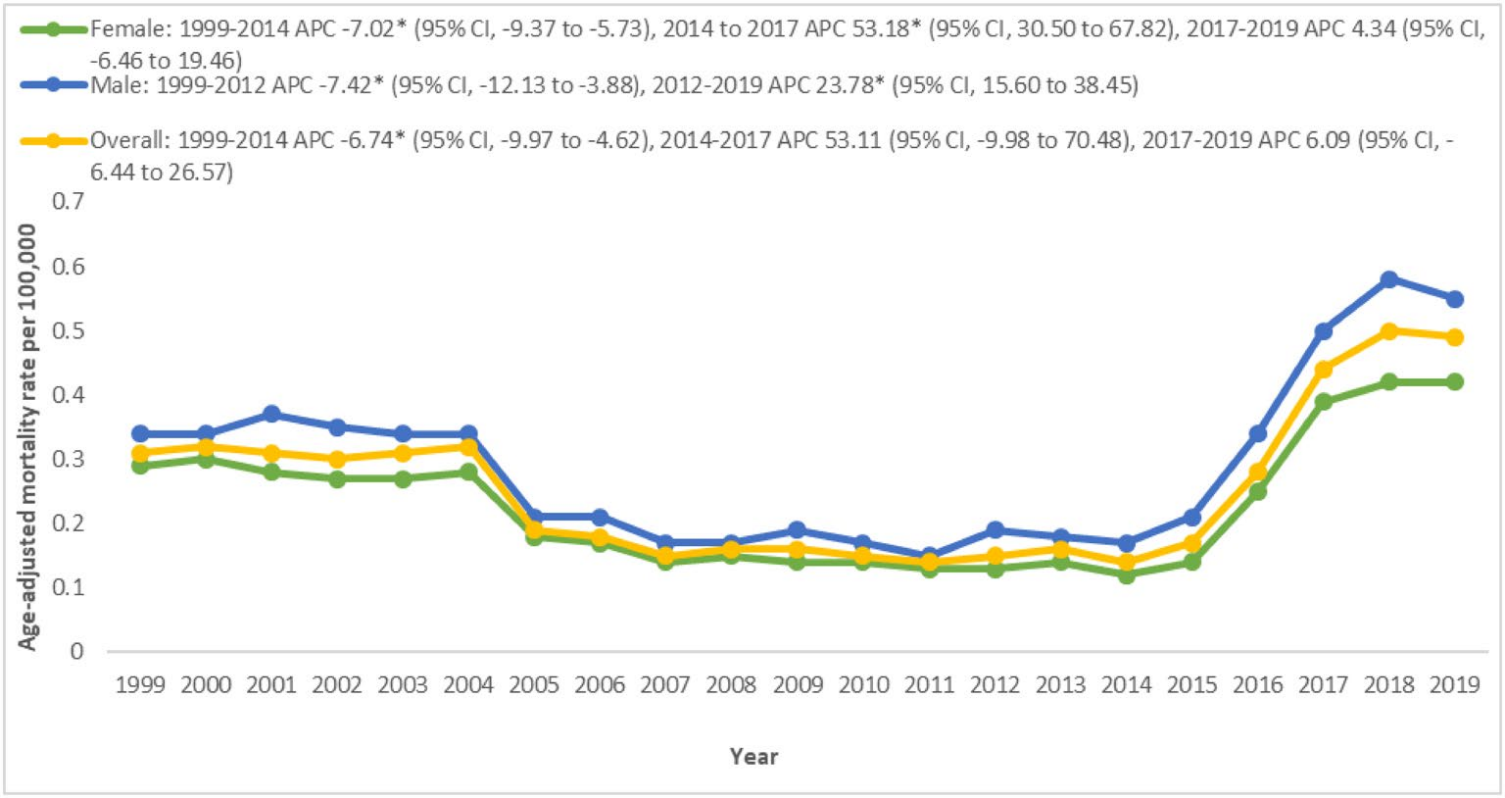
Overall and sex‐stratified ischemic stroke‐related AAMRs per 100,000 in patients with Type 2 diabetes mellitus in the United States, 1999–2019.

The overall AAMR for ischemic stroke in individuals with T2DM was 0.26 from 1999 to 2019. In 1999, the AAMR was 0.31, gradually declining to 0.14 in 2014 (APC: −6.74 [−9.97 to −4.62]). This downward trend was reversed by a sharp increase in AAMR to 0.44 in 2017 (APC: 53.11 [−9.98 to 70.48]). Subsequently, there was a consistent rise, reaching 0.49 in 2019 (APC: 6.09 [−6.44 to 26.57]) (Figure 1 and Table S2).

Upon evaluation of trends in ischemic stroke‐related mortality stratified by gender, 46.7% were males and 53% were females (Table S2). Overall, the AAMRs were observed to be consistently higher in males compared to females (overall AAMR male: 0.30; female: 0.23). In 1999, the AAMR for males was 0.34, which gradually decreased to 0.19 in 2012 (APC: −7.42 [−12.13 to −3.88]), followed by a steep rise to 0.55 in 2019 (APC: 23.78 [15.60 to 38.45]). For females, the AAMR was 0.29 in 1999, decreasing to 0.12 in 2014 (APC: −7.02 [−9.37 to −5.73]). After this decline, the AAMR surged to 0.39 in 2017 (APC: 53.18 [30.50–67.82]) and linearly increased to 0.42 in 2019 (APC: 4.34 [−6.46 to 19.46]) (Figure 1 and Table S3).

**FIGURE 2:**
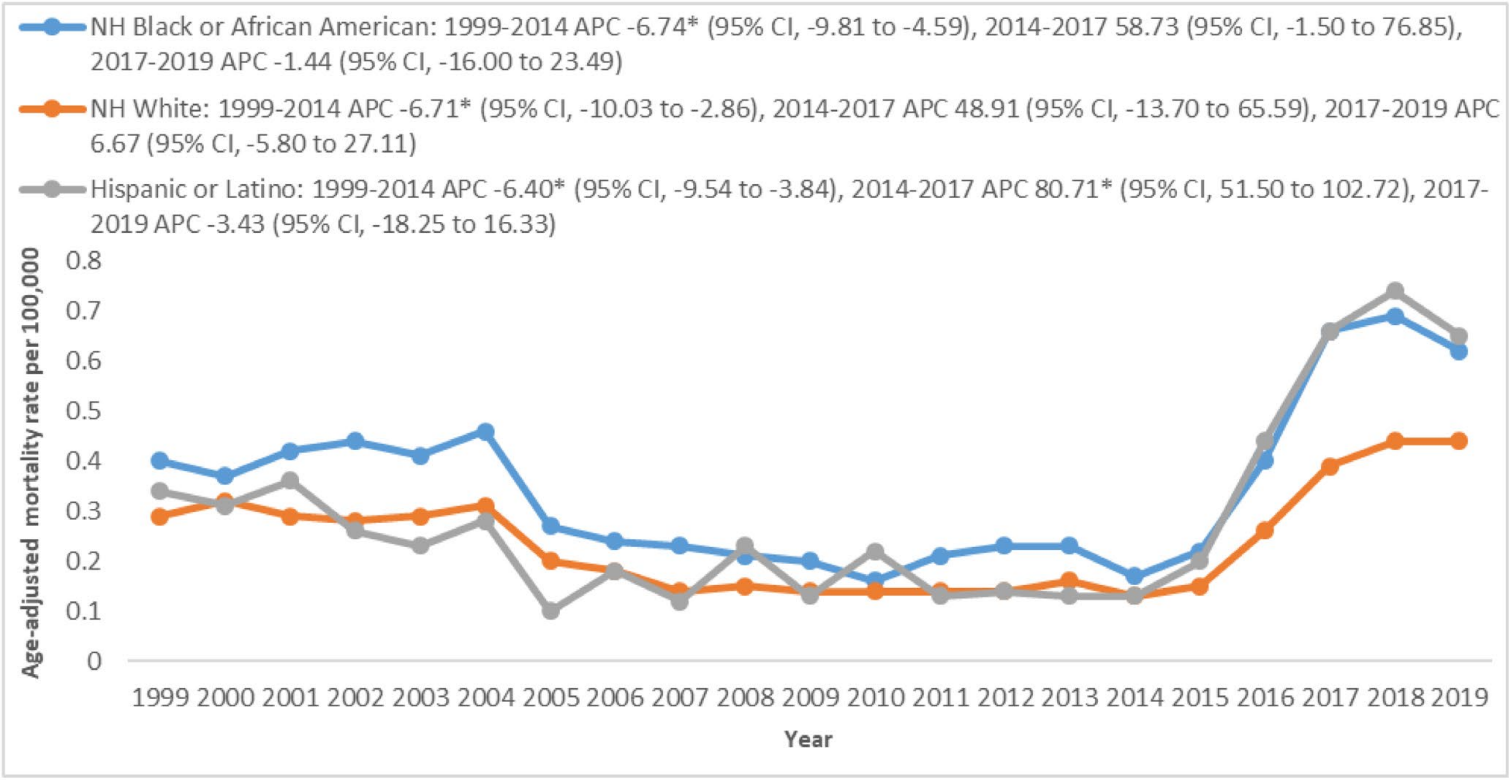
Ischemic stroke‐related AAMRs per 100,000 stratified by race in patients with Type 2 diabetes mellitus in the United States, 1999–2019.

When stratified by race/ethnicity, NH Whites accounted for 75.2% of the total deaths, NH Blacks accounted for 11.9%, and Hispanics accounted for 8.45% (Table S2). Among these groups, NH Blacks had the highest AAMRs, followed by Hispanics, and then NH Whites (overall AAMR NH Black: 0.34 [0.32–0.35]; Hispanics: 0.32 [0.30–0.33]; NH Whites: 0.24 [0.23–0.24]). In 1999, the AAMR for NH Blacks was 0.4, Hispanics was 0.34, and NH Whites was 0.29. The AAMRs for all three populations gradually declined from 1999 to 2014, reaching 0.17 in NH Blacks, 0.13 in Hispanics, and 0.13 in NH Whites (APC NH Blacks: −6.74 [−9.81 to −4.59]; Hispanics: −6.40 [−9.54 to −3.84]; NH Whites: −6.71 [−10.03 to −2.86]). This was followed by a sharp increase in AAMRs until 2017, reaching 0.66 in NH Blacks, 0.66 in Hispanics, and 0.39 in NH Whites (APC NH Blacks: 58.73 [−1.50 to 76.85]; Hispanics: 80.71 [51.50 to 102.72]; NH Whites: 48.91 [−13.70 to 65.59]). After 2017, the NH Whites experienced a consistent rise to 0.44 in 2019 (APC: 6.67 [−5.80 to 27.11]). However, in NH Blacks and Hispanics in 2019, the AAMRs decreased to 0.62 and 0.65, respectively (APC NH Blacks: −1.44 [−16.0 to 23.49]; Hispanics: −3.43 [−18.25 to 16.33]) (Figure 2 and Table S4).

**FIGURE 3:**
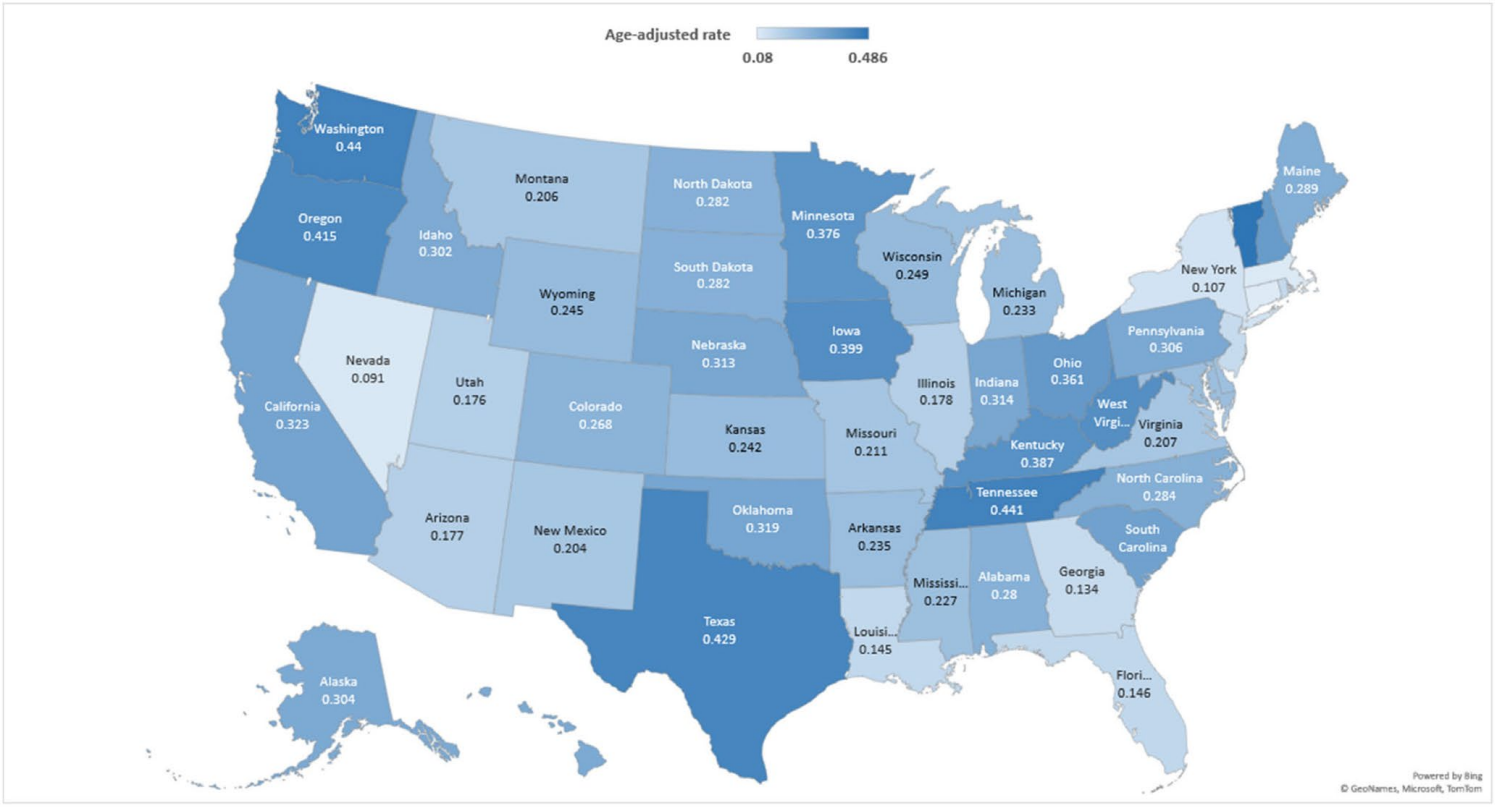
Geographical trending by state of ischemic stroke‐related AAMRs per 100,000 in patients with Type 2 diabetes mellitus in the United States, 1999–2019.

Across states, there is a notable variation in AAMR, ranging from 0.08 [0.06–0.1] in Connecticut to 0.49 [0.38–0.61] in Vermont. States falling within the top 90th percentile, such as Vermont, Tennessee, Washington, Texas, and Oregon, exhibited AAMRs nearly six times higher than those in the lower 10th percentile, including Connecticut, Massachusetts, Nevada, New York, Georgia, and New Jersey (Figure 3 and Table S5). During the study period, distinct regional patterns emerged, with the Western region (AAMR: 0.31 [0.30–0.32]) experiencing the highest mortality, followed by the Midwestern (AAMR: 0.28 [0.27–0.29]), Southern (AAMR: 0.28 [0.27–0.29]), and Northeastern regions (AAMR: 0.17 [0.16–0.17]) (Table S6).

**FIGURE 4:**
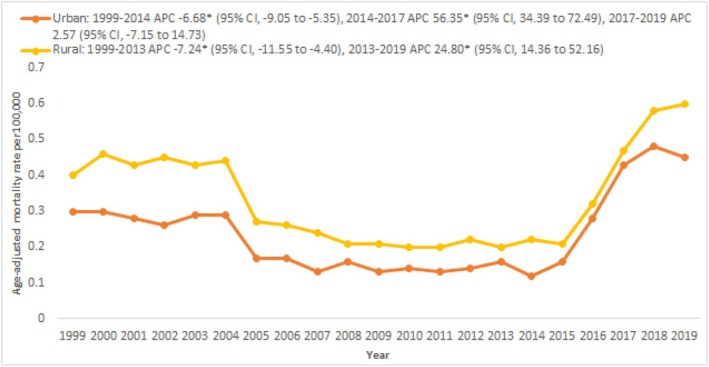
Ischemic stroke‐related AAMRs per 100,000 stratified by urbanisation status in patients with Type 2 diabetes mellitus in the United States, 1999–2019.

When categorised by rural–urban classification, 77.19% of total deaths occurred in urban areas, while 22.8% occurred in rural areas. Overall, the AAMRs were observed to be consistently higher in rural areas (AAMR: 0.33 [0.32–0.35]) as compared to urban areas (AAMR: 0.25 [0.24–0.25]). In 1999, the AAMR in rural areas was 0.4, which gradually decreased to 0.2 in 2013 (APC: −7.24 [−11.55 to −4.40]), followed by an increase to 0.6 in 2019 (APC: 24.80 [14.36–52.16]). In urban areas, the AAMR was 0.30 in 1999, which gradually declined to 0.12 in 2014 (APC: −6.68 [−9.05 to −5.35]). After this, there was a sharp increase to 0.43 in 2017 (APC: 56.35 [34.39–72.49]), followed by a consistent rise to 0.45 in 2019 (APC: 2.57 [−7.15 to 14.73]) (Figure 4 and Table S7).

## 4 Discussion

This 20‐year analysis of stroke‐related mortality in individuals with T2DM from the CDC WONDER database reveals several key findings. Firstly, there was a steady decline in mortality rates from 1999 to 2014, followed by a concerning reversal, with rates consistently rising through 2019. Secondly, this trend was observed across all racial groups, with NH Blacks reporting the highest AAMRs compared to other racial groups. Thirdly, non‐metropolitan areas consistently exhibited higher mortality rates than metropolitan areas throughout the 20‐year period. Additionally, significant variation was noted in mortality trends among states: those in the top 90th percentile (Vermont, Tennessee, Washington, Texas, and Oregon) exhibited mortality rates six times higher than those in the bottom 10th percentile (Connecticut, Massachusetts, Nevada, New York, Georgia, and New Jersey). These findings could have important implications for the prognosis of vascular abnormalities in patients with T2DM.

The sharp increase in AAMRs beginning around 2014 and accelerating between 2016 and 2019—observed across gender, racial, geographic, and urban–rural strata—represents a critical inflection point in the trends. Several possible factors may explain this reversal. First, while earlier decreases in AAMRs may be attributable to increased use of statins, antiplatelet agents, and improved management of hypertension and glycemia, the post‐2014 rise could reflect a stalling or reversal in those preventive efforts. This is supported by NHANES data, which show that, after 2014, fewer individuals with diabetes met glycemic and blood pressure targets, while cholesterol control remained stable [17]. Second, increasing rates of obesity and sedentary behaviour may have counteracted earlier gains in stroke prevention. The CDC reported that adult obesity prevalence rose to 42.4% by 2017–2018, with consistent trends across demographic subgroups [18]. This may have played a significant role in the post‐2016 escalation. Third, structural inequities in access to care—including uneven implementation of Medicaid expansion following the Affordable Care Act—may have worsened outcomes in certain regions and populations [19]. Fourth, while a change in ICD coding or death certification practices could theoretically contribute to a sudden rise in AAMRs, no major coding changes occurred between 2014 and 2019 that could account for the sharp inflection observed during this interval. Therefore, the post‐2014 uptick likely represents a true epidemiologic shift rather than a coding artifact. Nevertheless, this possibility cannot be fully excluded and remains an important area for future validation studies.

Although mortality rates have consistently been higher in non‐metropolitan areas compared to metropolitan areas, the recent trend shows a significant increase, highlighting the urgent need for targeted intervention. This surge in the rural diabetic population is associated with an increased risk of complications, exacerbated by the limited availability of medical care facilities, specialty services, and the often considerable distances from patients' homes [20, 21, 22]. A recent analysis of 41 states found that the prevalence of diabetes was higher in rural areas (14.3%) than in urban areas (11.2%), indicating a notable rural‐urban disparity [23]. These disparities likely stem from differences in population composition—such as older age, higher obesity rates, and greater concentrations of high‐risk sociodemographic groups in rural areas—and reflect a broader issue of insufficient research and resource allocation to meet the needs of rural diabetic populations [24, 25, 26, 27]. Further compounding this issue is the fact that diabetics in rural areas are significantly less likely to receive essential education on diabetes management and the importance of regular health check‐ups to achieve care goals [28, 29, 30]. For instance, a study analysing data from the 2019 Behavioural Risk Factor Surveillance System found that only 50.4% of rural residents reported having received diabetes self‐management education, compared to 55.5% of urban residents [31]. This lack of access to education and healthcare resources contributes to a notable difference in health outcomes between rural and urban diabetic patients. Further, a study of 45,279 diabetics found that patients in rural regions were significantly less likely to meet their D5 metric goals and had fewer outpatient and endocrinology visits (*p* < 0.001) compared to their urban counterparts [32].

Our analysis also reveals regional variations in stroke‐related mortality, with the Western regions bearing the highest burden compared to other U.S. regions. These disparities may be attributed to several factors, including a lack of basic healthcare education programs, insufficient healthcare facilities, differences in state‐based Medicaid regulations and medical expenditures, and spatial disparities in access to healthcare providers. Data from the National Plan and Provider Enumeration System (NPPES) and Medicare claims show lower spatial accessibility values for internal medicine physicians, family medicine physicians, nurses, and specialists in the Western regions compared to other U.S. regions [33]. Despite the well‐documented nature of these regional differences, efforts to address them through effective policymaking remain inadequate.

Furthermore, our analysis showed that diabetic men consistently had higher AAMRs throughout the study period compared to diabetic women. This finding aligns with some studies, although the literature remains conflicting. Some studies suggest that women with T2DM have an increased risk of complications and, consequently, higher stroke‐related mortality, while this trend appears less pronounced in men [34, 35]. Potential mechanisms for the increased mortality rates in women include reduced postmenopausal oestrogen levels, older age at T2DM onset, endothelial dysfunction, lower levels of anti‐inflammatory and neuroprotective hormones, and gradual brain damage over time [36, 37, 38, 39]. Our results diverge from the general observation that women with T2DM have higher mortality, which could be influenced by survival bias. The difference in average survival age between women and men increased from 5.1 years in 2019 to 5.8 years in 2021, which may partly explain the modest disparity observed between genders [40]. Given the ambiguity of these results and the potential for various explanations, larger population‐based studies are necessary to identify conclusive trends, confirm any apparent disparities, and explore the underlying mechanisms.

In addition, our data reveals that non‐Hispanic Black diabetics face the highest burden of ischemic stroke‐related mortality, followed by Hispanic and non‐Hispanic White individuals. Race and ethnicity, while socially constructed, are influenced by a range of societal, economic, and political factors. Racial inequities in the healthcare system are well documented, with Black individuals often receiving less adequate healthcare compared to other racial groups across the United States. These disparities in medical care may impact the prognosis of T2DM and contribute to observed trends in stroke‐related mortality. Data from the National Ambulatory Medical Care Survey (NAMCS) show poorer diabetes management among Black individuals, especially those in rural areas with limited access to routine clinical care [41]. This inadequate glycemic control is a known risk factor for ischemic stroke, which may partly explain the higher AAMRs observed in Black patients [42].

Several limitations should be noted in this study. First, our analysis focused on non‐Hispanic Black and non‐Hispanic White racial groups due to insufficient data for other racial groups, such as Asian, Hispanic, and American Indian populations. Including these groups could provide more comprehensive insights for policy‐making. Second, the database lacks critical clinical data, including vital signs, echocardiographic findings, and laboratory values. Third, while this study focuses on patients with T2DM, death certificates often do not differentiate between type 1 and type 2 diabetes, potentially leading to classification errors. Fourth, our inclusion criteria were based on deaths in which ischemic stroke and T2DM were listed as either underlying or contributing causes. This approach may introduce confounding from other primary cardiovascular causes of death—such as myocardial infarction—where T2DM is a major risk factor and stroke may only be a secondary event [43, 44]. Restricting the analysis to deaths where ischemic stroke is listed as the primary cause could reduce this potential confounding but would also risk underestimating the overall burden of stroke‐related mortality in diabetics. Finally, inaccuracies in determining the cause of death or incorrect ICD coding may result in misclassification and an underestimation of the actual cause of death.

## 5 Conclusion

In conclusion, our comprehensive analysis of ischemic stroke‐related mortality in individuals with T2DM over two decades reveals significant trends and disparities. We observed an initial decline in mortality rates until 2014, followed by a concerning reversal and consistent rise until 2019. NH Blacks exhibited the highest mortality rates, with substantial variations across racial and ethnic groups. Moreover, non‐metropolitan areas consistently reported higher mortality rates compared to metropolitan areas, signalling a need for targeted interventions in rural healthcare. Regional discrepancies further underscore the importance of addressing healthcare access and resource allocation. While our study sheds light on critical factors influencing stroke‐related mortality in diabetic individuals, further research is warranted to elucidate underlying mechanisms and inform effective preventive strategies tailored to diverse populations.

## Author Contributions

**Muhammad Moiz Nasir:** conceptualization, methodology, writing – review and editing, project administration. **Syed Husain Farhan:** conceptualization, investigation, writing – original draft. **Hasan Mushahid:** formal analysis, writing – original draft. **Syeda Ayesha Shah:** investigation, writing – original draft, writing – review and editing. **Muhammad Hamza Shuja:** investigation, writing – original draft, writing – review and editing. **Adam Bilal Khan:** formal analysis, writing – original draft. **Syed Hassaan Ali:** methodology, writing – reviewing and editing. **Syed Ahmed Farhan:** methodology, writing – original draft. **Azeem Hassan:** methodology, writing – original draft. **Jawad Ahmed:** formal analysis, writing – original draft, writing – review and editing. **Mohammad Hamza:** writing – original draft, writing – review and editing. **Javed Iqbal:** methodology.

## Conflicts of Interest

The authors declare no conflicts of interest.

## Supporting information


Appendix S1.

## Data Availability

The data that support the findings of this study are available on request from the corresponding author. The data are not publicly available due to privacy or ethical restrictions.
