# Supplementary material for: Ischemic Stroke Mortality in Type 2 Diabetes in the U.S.: National Trends and Demographic Disparities From 1999 to 2019
Source: Endocrinol Diabetes Metab. 2025 Jul 13;8(4):e70065. doi: 10.1002/edm2.70065 (PMC12256681; doi:10.1002/edm2.70065)
Supplement: Supplementary file 1 — Appendix S1. [file EDM2-8-e70065-s001.docx]

**Supplementary Data.**

|  | **Deaths** | | | |
| --- | --- | --- | --- | --- |
| **Year** | **Medical Facility** | **Home** | **Hospice** | **Nursing home/long term care** |
| **1999** | 389 | 106 | N/A | 349 |
| **2000** | 383 | 112 | N/A | 404 |
| **2001** | 410 | 95 | N/A | 348 |
| **2002** | 351 | 105 | N/A | 381 |
| **2003** | 398 | 115 | 0 | 352 |
| **2004** | 405 | 132 | N/A | 348 |
| **2005** | 314 | 87 | N/A | 166 |
| **2006** | 307 | 80 | N/A | 165 |
| **2007** | 255 | 65 | 18 | 124 |
| **2008** | 297 | 82 | 12 | 116 |
| **2009** | 261 | 88 | 18 | 128 |
| **2010** | 269 | 79 | 26 | 113 |
| **2011** | 240 | 89 | 31 | 130 |
| **2012** | 251 | 127 | 32 | 122 |
| **2013** | 278 | 111 | 32 | 134 |
| **2014** | 266 | 99 | 33 | 112 |
| **2015** | 273 | 142 | 43 | 165 |
| **2016** | 427 | 258 | 115 | 251 |
| **2017** | 701 | 410 | 172 | 399 |
| **2018** | 752 | 501 | 157 | 506 |
| **2019** | 648 | 540 | 177 | 522 |
| **Total** | **7875** | **3423** | **866** | **5335** |

**Table 1.** Ischemic Stroke–related Mortality, Stratified by Place of Death in patients with Type 2 Diabetes Mellitus in the United States, 1999 to 2019

|  | **Deaths** | | | | | | |
| --- | --- | --- | --- | --- | --- | --- | --- |
| **Year** | **Overall** | **Women** | **Men** | **NH White** | **NH Black or African American** | **Hispanic or Latino** | **Population** |
| 1999 | 863 | 502 | 361 | 707 | 83 | 46 | 279040168 |
| 2000 | 913 | 541 | 372 | 766 | 87 | 38 | 281421906 |
| 2001 | 874 | 481 | 393 | 710 | 96 | 47 | 284968955 |
| 2002 | 863 | 484 | 379 | 702 | 101 | 36 | 287625193 |
| 2003 | 894 | 508 | 386 | 735 | 97 | 36 | 290107933 |
| 2004 | 919 | 529 | 390 | 729 | 114 | 45 | 292805298 |
| 2005 | 590 | 341 | 249 | 471 | 71 | 21 | 295516599 |
| 2006 | 576 | 305 | 271 | 461 | 65 | 32 | 298379912 |
| 2007 | 475 | 265 | 210 | 367 | 60 | 26 | 301231207 |
| 2008 | 529 | 289 | 240 | 406 | 61 | 46 | 304093966 |
| 2009 | 516 | 273 | 243 | 409 | 58 | 31 | 306771529 |
| 2010 | 502 | 280 | 222 | 391 | 49 | 50 | 308745538 |
| 2011 | 503 | 273 | 230 | 394 | 59 | 33 | 311591917 |
| 2012 | 552 | 284 | 268 | 416 | 72 | 40 | 313914040 |
| 2013 | 585 | 312 | 273 | 448 | 75 | 36 | 316128839 |
| 2014 | 527 | 266 | 261 | 404 | 62 | 38 | 318857056 |
| 2015 | 645 | 325 | 320 | 470 | 74 | 69 | 321418820 |
| 2016 | 1093 | 539 | 554 | 762 | 142 | 140 | 323127513 |
| 2017 | 1738 | 880 | 858 | 1175 | 235 | 222 | 325719178 |
| 2018 | 2005 | 985 | 1020 | 1348 | 256 | 264 | 327167434 |
| 2019 | 1973 | 1008 | 965 | 1379 | 244 | 236 | 328239523 |
| **Total** | **18135** | **9670** | **8465** | **13650** | **2161** | **1532** | **6416872524** |

**Table 2.** Ischemic Stroke-related Mortality, Stratified by Sex and Race in Patients with Type 2 Diabetes Mellitus in the United States, 1999-2019

|  | **Age-Adjusted Rate (95% CI)** | | |
| --- | --- | --- | --- |
| **Year** | **Overall** | **Men** | **Women** |
| **1999** | 0.31 | 0.34 | 0.29 |
|  | (0.29 - 0.33) | (0.31 - 0.38) | (0.26 - 0.31) |
| **2000** | 0.32 | 0.34 | 0.3 |
|  | (0.30 - 0.34) | (0.31 - 0.38) | (0.28 - 0.33) |
| **2001** | 0.31 | 0.37 | 0.28 |
|  | (0.29 - 0.33) | (0.33 - 0.41) | (0.25 - 0.30) |
| **2002** | 0.3 | 0.35 | 0.27 |
|  | (0.28 - 0.32) | (0.31 - 0.38) | (0.24 - 0.29) |
| **2003** | 0.31 | 0.34 | 0.27 |
|  | (0.29 - 0.33) | (0.30 - 0.37) | (0.25 - 0.30) |
| **2004** | 0.32 | 0.34 | 0.28 |
|  | (0.30 - 0.34) | (0.30 - 0.37) | (0.26 - 0.31) |
| **2005** | 0.19 | 0.21 | 0.18 |
|  | (0.17 - 0.20) | (0.18 - 0.23) | (0.16 - 0.20) |
| **2006** | 0.18 | 0.21 | 0.17 |
|  | (0.17 - 0.20) | (0.19 - 0.24) | (0.15 - 0.19) |
| **2007** | 0.15 | 0.17 | 0.14 |
|  | (0.13 - 0.16) | (0.15 - 0.20) | (0.13 - 0.16) |
| **2008** | 0.16 | 0.17 | 0.15 |
|  | (0.15 - 0.17) | (0.15 - 0.20) | (0.13 - 0.17) |
| **2009** | 0.16 | 0.19 | 0.14 |
|  | (0.14 - 0.17) | (0.16 - 0.21) | (0.12 - 0.15) |
| **2010** | 0.15 | 0.17 | 0.14 |
|  | (0.13 - 0.16) | (0.15 - 0.19) | (0.12 - 0.15) |
| **2011** | 0.14 | 0.15 | 0.13 |
|  | (0.13 - 0.15) | (0.13 - 0.17) | (0.11 - 0.15) |
| **2012** | 0.15 | 0.19 | 0.13 |
|  | (0.14 - 0.16) | (0.17 - 0.21) | (0.11 - 0.15) |
| **2013** | 0.16 | 0.18 | 0.14 |
|  | (0.14 - 0.17) | (0.15 - 0.20) | (0.12 - 0.16) |
| **2014** | 0.14 | 0.17 | 0.12 |
|  | (0.13 - 0.16) | (0.15 - 0.19) | (0.10 - 0.13) |
| **2015** | 0.17 | 0.21 | 0.14 |
|  | (0.16 - 0.18) | (0.19 - 0.23) | (0.13 - 0.16) |
| **2016** | 0.28 | 0.34 | 0.25 |
|  | (0.26 - 0.30) | (0.31 - 0.37) | (0.23 - 0.27) |
| **2017** | 0.44 | 0.5 | 0.39 |
|  | (0.42 - 0.46) | (0.47 - 0.54) | (0.36 - 0.42) |
| **2018** | 0.5 | 0.58 | 0.42 |
|  | (0.48 - 0.52) | (0.54 - 0.61) | (0.39 - 0.45) |
| **2019** | 0.49 | 0.55 | 0.42 |
|  | (0.47 - 0.51) | (0.52 - 0.59) | (0.40 - 0.45) |
| **Overall AAMR** | **0.26** | **0.3** | **0.23** |
|  | **(0.26 - 0.27)** | **(0.30 - 0.31)** | **(0.22 - 0.23)** |

**Table 3.** Overall and Sex-Stratified Ischemic Stroke–related Age-Adjusted Mortality Rates per 100,000 in patients with Type 2 Diabetes Mellitus in the United States, 1999 to 2019

|  | **Age-Adjusted Rate (95% CI)** | | |
| --- | --- | --- | --- |
| **Year** | **NH Black or African American** | **NH White** | **Hispanic or Latino** |
| **1999** | 0.4 | 0.29 | 0.34 |
|  | (0.32 - 0.50) | (0.27 - 0.32) | (0.25 - 0.46) |
| **2000** | 0.37 | 0.32 | 0.31 |
|  | (0.30 - 0.46) | (0.30 - 0.35) | (0.22 - 0.43) |
| **2001** | 0.42 | 0.29 | 0.36 |
|  | (0.34 - 0.51) | (0.27 - 0.31) | (0.26 - 0.48) |
| **2002** | 0.44 | 0.28 | 0.26 |
|  | (0.36 - 0.53) | (0.26 - 0.30) | (0.18 - 0.36) |
| **2003** | 0.41 | 0.29 | 0.23 |
|  | (0.33 - 0.50) | (0.27 - 0.32) | (0.16 - 0.32) |
| **2004** | 0.46 | 0.31 | 0.28 |
|  | (0.37 - 0.54) | (0.29 - 0.33) | (0.20 - 0.38) |
| **2005** | 0.27 | 0.2 | 0.1 |
|  | (0.21 - 0.34) | (0.18 - 0.21) | (0.06 - 0.16) |
| **2006** | 0.24 | 0.18 | 0.18 |
|  | (0.18 - 0.31) | (0.16 - 0.19) | (0.12 - 0.26) |
| **2007** | 0.23 | 0.14 | 0.12 |
|  | (0.18 - 0.30) | (0.13 - 0.15) | (0.08 - 0.18) |
| **2008** | 0.21 | 0.15 | 0.23 |
|  | (0.16 - 0.28) | (0.13 - 0.16) | (0.17 - 0.31) |
| **2009** | 0.2 | 0.14 | 0.13 |
|  | (0.15 - 0.26) | (0.13 - 0.16) | (0.08 - 0.19) |
| **2010** | 0.16 | 0.14 | 0.22 |
|  | (0.12 - 0.22) | (0.13 - 0.16) | (0.16 - 0.29) |
| **2011** | 0.21 | 0.14 | 0.13 |
|  | (0.16 - 0.28) | (0.12 - 0.15) | (0.09 - 0.19) |
| **2012** | 0.23 | 0.14 | 0.14 |
|  | (0.18 - 0.29) | (0.13 - 0.15) | (0.10 - 0.20) |
| **2013** | 0.23 | 0.16 | 0.13 |
|  | (0.18 - 0.30) | (0.14 - 0.17) | (0.09 - 0.19) |
| **2014** | 0.17 | 0.13 | 0.13 |
|  | (0.13 - 0.22) | (0.12 - 0.14) | (0.09 - 0.18) |
| **2015** | 0.22 | 0.15 | 0.2 |
|  | (0.17 - 0.28) | (0.14 - 0.17) | (0.15 - 0.26) |
| **2016** | 0.4 | 0.26 | 0.44 |
|  | (0.33 - 0.47) | (0.24 - 0.28) | (0.37 - 0.52) |
| **2017** | 0.66 | 0.39 | 0.66 |
|  | (0.58 - 0.75) | (0.36 - 0.41) | (0.57 - 0.75) |
| **2018** | 0.69 | 0.44 | 0.74 |
|  | (0.61 - 0.78) | (0.41 - 0.46) | (0.64 - 0.83) |
| **2019** | 0.62 | 0.44 | 0.65 |
|  | (0.54 - 0.70) | (0.41 - 0.46) | (0.56 - 0.73) |
| **Overall AAMR** | **0.34** | **0.24** | **0.32** |
|  | **(0.32 - 0.35)** | **(0.23 - 0.24)** | **(0.30 - 0.33)** |

**Table 4.** Ischemic Stroke–related Age-Adjusted Mortality Rates per 100,000, Stratified by Race in patients with Type 2 Diabetes Mellitus in the United States, 1999 to 2019

| **State** | **Age-adjusted rate per 100,000 (95% CI)** |
| --- | --- |
| **Connecticut** | 0.08 |
|  | (0.063 - 0.101) |
| **Massachusetts** | 0.084 |
|  | (0.070 - 0.098) |
| **Nevada** | 0.091 |
|  | (0.066 - 0.121) |
| **New York** | 0.107 |
|  | (0.098 - 0.117) |
| **Georgia** | 0.134 |
|  | (0.117 - 0.152) |
| **New Jersey** | 0.134 |
|  | (0.118 - 0.150) |
| **Rhode Island** | 0.14 |
|  | (0.097 - 0.194) |
| **Louisiana** | 0.145 |
|  | (0.120 - 0.169) |
| **Florida** | 0.146 |
|  | (0.136 - 0.156) |
| **Utah** | 0.176 |
|  | (0.137 - 0.221) |
| **Arizona** | 0.177 |
|  | (0.154 - 0.199) |
| **Illinois** | 0.178 |
|  | (0.162 - 0.194) |
| **New Mexico** | 0.204 |
|  | (0.162 - 0.253) |
| **Montana** | 0.206 |
|  | (0.154 - 0.270) |
| **Virginia** | 0.207 |
|  | (0.185 - 0.229) |
| **Missouri** | 0.211 |
|  | (0.186 - 0.235) |
| **Delaware** | 0.226 |
|  | (0.166 - 0.300) |
| **Mississippi** | 0.227 |
|  | (0.190 - 0.264) |
| **Maryland** | 0.231 |
|  | (0.204 - 0.258) |
| **Michigan** | 0.233 |
|  | (0.213 - 0.253) |
| **Arkansas** | 0.235 |
|  | (0.199 - 0.271) |
| **Kansas (20)** | 0.242 |
|  | (0.204 - 0.279) |
| **Wyoming** | 0.245 |
|  | (0.161 - 0.356) |
| **Wisconsin** | 0.249 |
|  | (0.223 - 0.275) |
| **Colorado** | 0.268 |
|  | (0.234 - 0.302) |
| **Alabama** | 0.28 |
|  | (0.249 - 0.311) |
| **North Dakota** | 0.282 |
|  | (0.209 - 0.373) |
| **South Dakota** | 0.282 |
|  | (0.215 - 0.363) |
| **North Carolina** | 0.284 |
|  | (0.261 - 0.308) |
| **Maine** | 0.289 |
|  | (0.233 - 0.344) |
| **Idaho** | 0.302 |
|  | (0.244 - 0.370) |
| **Alaska** | 0.304 |
|  | (0.195 - 0.452) |
| **Hawaii** | 0.304 |
|  | (0.244 - 0.364) |
| **Pennsylvania** | 0.306 |
|  | (0.287 - 0.325) |
| **Nebraska** | 0.313 |
|  | (0.259 - 0.367) |
| **Indiana** | 0.314 |
|  | (0.285 - 0.343) |
| **Oklahoma** | 0.319 |
|  | (0.281 - 0.356) |
| **California** | 0.323 |
|  | (0.310 - 0.336) |
| **South Carolina** | 0.332 |
|  | (0.297 - 0.368) |
| **New Hampshire** | 0.352 |
|  | (0.285 - 0.418) |
| **Ohio** | 0.361 |
|  | (0.339 - 0.384) |
| **Minnesota** | 0.376 |
|  | (0.342 - 0.411) |
| **Kentucky** | 0.387 |
|  | (0.347 - 0.426) |
| **West Virginia** | 0.396 |
|  | (0.341 - 0.452) |
| **Iowa** | 0.399 |
|  | (0.356 - 0.443) |
| **Oregon** | 0.415 |
|  | (0.372 - 0.457) |
| **Texas** | 0.429 |
|  | (0.410 - 0.449) |
| **Washington** | 0.44 |
|  | (0.405 - 0.474) |
| **Tennessee** | 0.441 |
|  | (0.406 - 0.476) |
| **Vermont** | 0.486 |
|  | (0.381 - 0.610) |

**Table 5.** Ischemic Stroke–related Age-Adjusted Mortality Rates per 100,000, Stratified by States in patients with Type 2 Diabetes Mellitus in the United States, 1999 to 2019

|  | **Age-Adjusted Rate (95% CI)** | | | |
| --- | --- | --- | --- | --- |
| **Year** | **Northeast** | **Midwest** | **South** | **West** |
| **1999** | 0.22 | 0.39 | 0.31 | 0.31 |
|  | (0.18 - 0.25) | (0.34 - 0.44) | (0.27 - 0.35) | (0.27 - 0.36) |
| **2000** | 0.3 | 0.38 | 0.31 | 0.31 |
|  | (0.26 - 0.35) | (0.33 - 0.43) | (0.28 - 0.35) | (0.26 - 0.36) |
| **2001** | 0.23 | 0.35 | 0.33 | 0.31 |
|  | (0.19 - 0.27) | (0.31 - 0.40) | (0.29 - 0.37) | (0.26 - 0.35) |
| **2002** | 0.23 | 0.34 | 0.32 | 0.27 |
|  | (0.20 - 0.27) | (0.29 - 0.38) | (0.29 - 0.36) | (0.23 - 0.31) |
| **2003** | 0.23 | 0.33 | 0.33 | 0.33 |
|  | (0.19 - 0.27) | (0.29 - 0.38) | (0.29 - 0.36) | (0.28 - 0.37) |
| **2004** | 0.21 | 0.32 | 0.34 | 0.35 |
|  | (0.18 - 0.25) | (0.27 - 0.36) | (0.31 - 0.38) | (0.30 - 0.40) |
| **2005** | 0.15 | 0.25 | 0.21 | 0.16 |
|  | (0.12 - 0.19) | (0.21 - 0.29) | (0.18 - 0.24) | (0.13 - 0.20) |
| **2006** | 0.13 | 0.23 | 0.2 | 0.16 |
|  | (0.11 - 0.17) | (0.20 - 0.27) | (0.18 - 0.23) | (0.13 - 0.19) |
| **2007** | 0.1 | 0.19 | 0.17 | 0.13 |
|  | (0.08 - 0.13) | (0.16 - 0.22) | (0.15 - 0.20) | (0.11 - 0.17) |
| **2008** | 0.1 | 0.2 | 0.16 | 0.2 |
|  | (0.08 - 0.13) | (0.17 - 0.23) | (0.14 - 0.19) | (0.16 - 0.23) |
| **2009** | 0.12 | 0.2 | 0.14 | 0.15 |
|  | (0.10 - 0.15) | (0.17 - 0.23) | (0.12 - 0.17) | (0.12 - 0.18) |
| **2010** | 0.12 | 0.16 | 0.16 | 0.15 |
|  | (0.09 - 0.14) | (0.13 - 0.19) | (0.13 - 0.18) | (0.12 - 0.18) |
| **2011** | 0.1 | 0.17 | 0.14 | 0.18 |
|  | (0.08 - 0.13) | (0.14 - 0.19) | (0.11 - 0.16) | (0.15 - 0.21) |
| **2012** | 0.12 | 0.17 | 0.16 | 0.2 |
|  | (0.09 - 0.14) | (0.14 - 0.20) | (0.14 - 0.18) | (0.16 - 0.23) |
| **2013** | 0.12 | 0.18 | 0.17 | 0.17 |
|  | (0.09 - 0.15) | (0.15 - 0.22) | (0.15 - 0.20) | (0.14 - 0.19) |
| **2014** | 0.12 | 0.16 | 0.14 | 0.16 |
|  | (0.10 - 0.15) | (0.13 - 0.19) | (0.12 - 0.16) | (0.13 - 0.19) |
| **2015** | 0.09 | 0.18 | 0.17 | 0.21 |
|  | (0.07 - 0.12) | (0.15 - 0.21) | (0.15 - 0.20) | (0.18 - 0.24) |
| **2016** | 0.16 | 0.28 | 0.31 | 0.35 |
|  | (0.13 - 0.19) | (0.24 - 0.31) | (0.28 - 0.34) | (0.31 - 0.39) |
| **2017** | 0.19 | 0.42 | 0.44 | 0.68 |
|  | (0.16 - 0.22) | (0.38 - 0.47) | (0.41 - 0.48) | (0.62 - 0.74) |
| **2018** | 0.28 | 0.46 | 0.5 | 0.74 |
|  | (0.24 - 0.32) | (0.41 - 0.50) | (0.46 - 0.53) | (0.68 - 0.79) |
| **2019** | 0.25 | 0.4 | 0.58 | 0.57 |
|  | (0.22 - 0.29) | (0.36 - 0.44) | (0.54 - 0.62) | (0.52 - 0.62) |
| **Overall AAMR** | **0.17** | **0.28** | **0.28** | **0.31** |
|  | **(0.16 - 0.17)** | **(0.27 - 0.29)** | **(0.27 - 0.29)** | **(0.30 - 0.32)** |

**Table 6.** Ischemic Stroke–related Age-Adjusted Mortality Rates per 100,000, Stratified by Census Region in patients with Type 2 Diabetes Mellitus in the United States, 1999 to 2019

|  | **Age-Adjusted Rate (95% CI)** | |
| --- | --- | --- |
| **Year** | **Urban** | **Rural** |
| **1999** | 0.3 | 0.4 |
|  | (0.27 - 0.32) | (0.35 - 0.45) |
| **2000** | 0.3 | 0.46 |
|  | (0.27 - 0.32) | (0.40 - 0.52) |
| **2001** | 0.28 | 0.43 |
|  | (0.26 - 0.31) | (0.37 - 0.48) |
| **2002** | 0.26 | 0.45 |
|  | (0.24 - 0.28) | (0.39 - 0.50) |
| **2003** | 0.29 | 0.43 |
|  | (0.27 - 0.31) | (0.37 - 0.48) |
| **2004** | 0.29 | 0.44 |
|  | (0.27 - 0.31) | (0.39 - 0.50) |
| **2005** | 0.17 | 0.27 |
|  | (0.15 - 0.18) | (0.22 - 0.31) |
| **2006** | 0.17 | 0.26 |
|  | (0.15 - 0.18) | (0.22 - 0.30) |
| **2007** | 0.13 | 0.24 |
|  | (0.11 - 0.14) | (0.20 - 0.28) |
| **2008** | 0.16 | 0.21 |
|  | (0.14 - 0.17) | (0.17 - 0.24) |
| **2009** | 0.13 | 0.21 |
|  | (0.12 - 0.15) | (0.18 - 0.25) |
| **2010** | 0.14 | 0.2 |
|  | (0.12 - 0.15) | (0.17 - 0.24) |
| **2011** | 0.13 | 0.2 |
|  | (0.12 - 0.15) | (0.16 - 0.24) |
| **2012** | 0.14 | 0.22 |
|  | (0.12 - 0.15) | (0.18 - 0.27) |
| **2013** | 0.16 | 0.2 |
|  | (0.15 - 0.18) | (0.16 - 0.23) |
| **2014** | 0.12 | 0.22 |
|  | (0.11 - 0.13) | (0.18 - 0.25) |
| **2015** | 0.16 | 0.21 |
|  | (0.15 - 0.18) | (0.17 - 0.24) |
| **2016** | 0.28 | 0.32 |
|  | (0.26 - 0.30) | (0.28 - 0.37) |
| **2017** | 0.43 | 0.47 |
|  | (0.40 - 0.45) | (0.41 - 0.52) |
| **2018** | 0.48 | 0.58 |
|  | (0.46 - 0.51) | (0.52 - 0.64) |
| **2019** | 0.45 | 0.6 |
|  | (0.43 - 0.47) | (0.54 - 0.66) |
| **Overall AAMR** | **0.25** | **0.33** |
|  | **(0.24 - 0.25)** | **(0.32 - 0.35)** |

**Table 7.** Ischemic Stroke–related Age-Adjusted Mortality Rates per 100,000, Stratified by Urbanization Status in patients with Type 2 Diabetes Mellitus in the United States, 1999 to 2019
